# Influence of in-line microfilters on systemic inflammation in adult critically ill patients: a prospective, randomized, controlled open-label trial

**DOI:** 10.1186/s13613-015-0080-x

**Published:** 2015-11-04

**Authors:** Ilse Gradwohl-Matis, Andreas Brunauer, Daniel Dankl, Elisabeth Wirthel, Ingeborg Meburger, Angela Bayer, Michaela Mandl, Martin W. Dünser, Wilhelm Grander

**Affiliations:** Interdisciplinary Intensive Care Units, Department of Anesthesiology, Perioperative and General Intensive Care, Salzburg University Hospital and Paracelsus Private Medical University, Müllner Hauptstrasse 48, 5020 Salzburg, Austria; Pharmacy of the Bundesland Salzburg, Salzburg, Austria; Department of Internal Medicine, General Hospital Hall in Tirol, Hall in Tirol, Austria

**Keywords:** In-line microfilter, Adults, Critically ill, Systemic inflammation, C-Reactive protein

## Abstract

**Background:**

In critically ill children, in-line microfilters may reduce the incidence of the systemic inflammatory response syndrome (SIRS), the overall complication and organ dysfunction rate. No data on the use of in-line microfilters exist in critically ill adults.

**Methods:**

In this prospective, randomized, controlled open-label study, we evaluated the influence of in-line microfilters on systemic immune activation in 504 critically ill adults with a central venous catheter in place and an expected length of stay in the intensive care unit >24 h. Patients were randomized to have in-line microfilters placed into all intravenous lines (intervention group) or usual care (control group). The primary endpoint was the number of intensive care unit days with SIRS. Secondary endpoints were the incidence of SIRS, SIRS criteria per day, duration of invasive mechanical ventilation, intensive care unit length of stay, the incidence of acute lung injury, maximum C-reactive protein, maximum white blood cell count, incidence of new candida and/or central-line-associated bloodstream infections, incidence of new thromboembolic complications, cumulative insulin requirements and presence of hyper- or hypoglycemia.

**Results:**

The study groups did not differ in any baseline variable. There was no difference in the number of days in the intensive care unit with SIRS between microfilter and control patients [2 (0.8–4.7) vs. 1.8 (0.7–4.4), *p* = 0.62]. Except for a higher incidence of SIRS in microfilter patients (99.6 vs. 96.8 %, *p* = 0.04), no difference between the groups was observed in any secondary outcome parameter. Results did not change when only patients with an intensive care unit length of stay of greater than 7 days were included in the analysis. The rate of adverse events was comparable between microfilter and control patients. In two patients allocated to the microfilter group, the study intervention was discontinued for technical reasons. Use of in-line microfilters was associated with additional costs.

**Conclusions:**

The use of in-line microfilters failed to modulate systemic inflammation and clinical outcome parameters in critically ill adults.

Trial registration: Clinical Trials NCT01534390

## Background

Injection or infusion of drugs and fluids releases microparticles into the bloodstream [1, 2]. Particulate contamination arises from manufacture, packaging and transport of solutions and drugs [3] or drug incompatibility reactions [4]. These particles may stimulate the immune system and cause organ damage, thus aggravating the underlying disease [5]. As a pro-inflammatory state commonly occurs during critical illness [6], patients in the intensive care unit (ICU) may be specifically vulnerable to particle infusion and additional immune stimulation [3]. Systemic stimulation of the immune system in critically ill patients is a risk factor for multiple organ dysfunction and death [6]. Mechanisms of particle-induced organ damage are a mechanical blockage of microvessels [3], the activation of platelets and neutrophilic granulocytes with the generation of occlusive micro-thrombi [3] and the formation of foreign body granulomas [7–9].

In-line microfilters are placed in the infusion lines and were first used in the 1960s to avoid particle exposition of patients during intravenous drug therapies [7, 10]. Since then, in-line microfilters have repeatedly been shown to prevent particles from being introduced into the bloodstream [11]. In-line microfilters with small enough pores may even retain certain microorganisms, such as large bacteria and fungal spores, endotoxins and air [12]. In 807 critically ill children, use of in-line microfilters was associated with a reduction in the overall complication rate and incidence of the systemic inflammatory response syndrome (SIRS) [13]. The same study group showed that in-line filtration had beneficial effects on the preservation of hematologic, renal and respiratory function in critically ill children [14]. While other authors confirmed these positive results in critically ill children and neonates [9, 15, 16], no data on the use of in-line microfilters exist in critically ill adults.

In this prospective, randomized, controlled open-label trial, the influence of in-line microfilters on systemic immune activation was evaluated. We hypothesized that the use of in-line microfilters reduces the number of days with SIRS.

## Methods

This study was designed as a single-center, prospective, randomized, controlled, open-label trial. It was conducted in a 22-bed interdisciplinary ICU of a tertiary care university teaching hospital during the time from April 2012 to August 2013. The study protocol was approved by the Ethics Committee of the Land Salzburg (415-E/1442/7-2012). Written informed consent was obtained from all patients or their legal representatives. The trial was registered at the Clinical Trials database of the US National Institutes of Health (trial registration number, NCT01534390; https://clinicaltrials.gov/ct2/show/NCT01534390; date of registration: February 9, 2012).

### Patients and randomization

All critically ill patients older than 18 years with an expected length of stay in the ICU > 24 h and a central venous catheter in place or one placed within the first 24 h after ICU admission were eligible for study entry. Patients who did not meet any exclusion criteria were randomly assigned into a study and a control group using a computer-generated randomization list. Age < 18 years, pregnancy, neutropenia (< 1.5 G/L) or known immunosuppression, limited intensive care, inclusion into another clinical trial, and refusal of written informed consent were considered exclusion criteria. In case patients were re-admitted to the ICU, they were enrolled in the study only during their first ICU stay.

### Study intervention

**Fig. 1:**
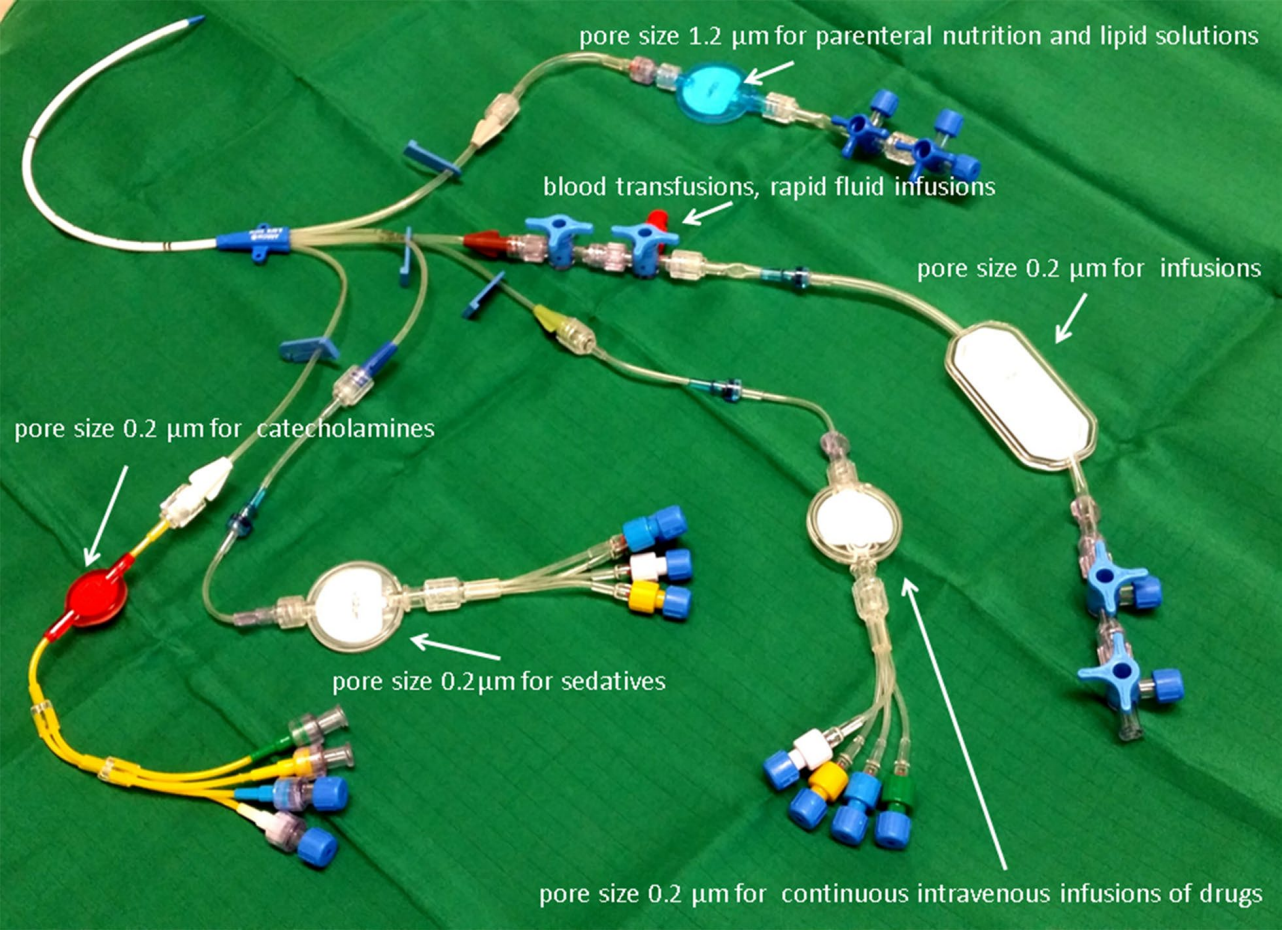
Schematic overview of different sized and colored in-line filters on a central venous catheter

In patients allocated to the study group, in-line microfilters were placed into all intravenous lines as soon as possible following ICU admission. Patients assigned to the control group received usual care. Study group allocation was retained for the entire ICU stay. The in-line microfilters used (MedCare; Oberwang, Austria) had a pore size of 0.2 or 1.2 µm and consisted of polyethersulfone membranes with a low adsorption profile (for maximum transmission of proteins and extensive drug compatibility). All products used were approved for intravenous therapy and CE-certified (Supor IV Filter^®^; Pall Corporation, Port Washington, NY, USA). According to an institutional algorithm, in-line microfilters were introduced into each lumen of venous catheters. 0.2 µm pore size positively charged filters were used for aqueous solutions and 1.2 µm pore size filters for infusion of lipid-containing admixtures. Because of the small pore size and the resulting reduction of flow, all intravenous solutions were administered by infusion or syringe pumps. In emergency cases, when rapid fluid infusions or blood transfusions were required, in-line microfilters could be bypassed by administering fluids and blood products via a three-way cock placed into the infusion line following the microfilters. According to an institutionally standardized colour scheme, different infusion lines were used for special medications to prevent drug incompatibility (Fig. 1). 1.2 µm filters were replaced daily, while 0.2 µm filters were replaced following 72 h of regular use.

### Data collection and definitions

At study entry, demographic data, comorbidities and the admission diagnosis were collected. The presence of SIRS, as defined by the ACCP/SCCM criteria [17], was documented daily-based electronic data documentation (MetaVision^®^; IMDSoft, Tel Aviv, Israel) at 1-min intervals of heart rate, core temperature and respiratory rate, as well as once daily measurement of the white blood cell count. During the study period, patients were screened for specific therapies (blood transfusion, vasopressor therapy, invasive mechanical ventilation, renal replacement or extracorporeal membrane oxygenation therapy), occurrence of acute lung injury or the acute respiratory distress syndrome [18], candida bloodstream infection, central-line-associated bloodstream infection, any new thromboembolic complication, presence of hyper- (blood glucose level > 180 mg/dL) or hypoglycemia (blood glucose level < 75 mg/dL), as well as the need for insulin therapy and cumulative insulin requirements during the ICU stay. At ICU discharge, the maximum C-reactive protein serum concentration and white blood cell count during the ICU stay were documented. In addition, duration of invasive mechanical ventilation, length of ICU stay, and the survival status at ICU discharge were collected. In patients randomized to the in-line microfilter group, additional costs arising from the use of in-line microfilters were calculated.

### Primary and secondary study endpoints

The primary study endpoint was the number of ICU days with SIRS. Secondary study endpoints were the incidence of SIRS, the number of SIRS criteria per day with the syndrome, the duration of invasive mechanical ventilation, the length of stay in the ICU, the incidence of acute lung injury/acute respiratory distress syndrome, the maximum C-reactive protein serum concentration, the maximum white blood cell count, the incidence of new candida and/or central-line-associated bloodstream infections, the incidence of new thromboembolic complications, cumulative insulin requirements and presence of hyper- or hypoglycemia.

### Statistical analysis

We estimated that a sample size of 504 patients (252 per study group) would provide an 80 % power to detect a reduction in the number of ICU days with SIRS from 2 ± 2 days by 0.5 days to 1.5 ± 2 days at a two-sided alpha-error of 5 %. All statistical analyses were based on the intention-to-treat principle. No blinding of outcome assessment was performed. Normality distribution of the study variables was tested with Shapiro–Wilks tests and was approximately fulfilled in all study parameters. Comparisons between the study and control group were performed using the Mann–Whitney *U*- or Fisher’s Exact test, as appropriate. In a post hoc analysis, we repeated the comparisons of primary and secondary outcome parameters between study and control patients in a selected population of patients with an ICU length of stay >7 days. All tests were two-sided, and a *p* value <0.05 was considered to indicate statistical significance. Quantitative data are expressed as median values with interquartile ranges, qualitative data as numbers and percentages. The IBM SPSS Statistics software was used for statistical analysis (IBM SPSS Statistics 20; Erlangen, Germany).

## Results

**Fig. 2:**
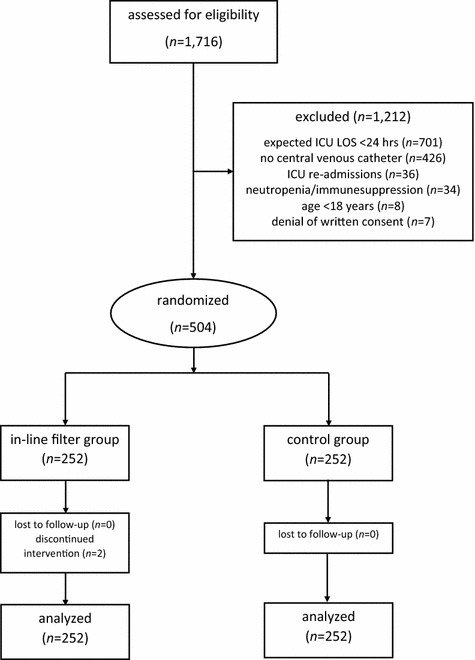
Overview of patient enrollment. *ICU* intensive care unit, *LOS* length of stay

**Fig. 3:**
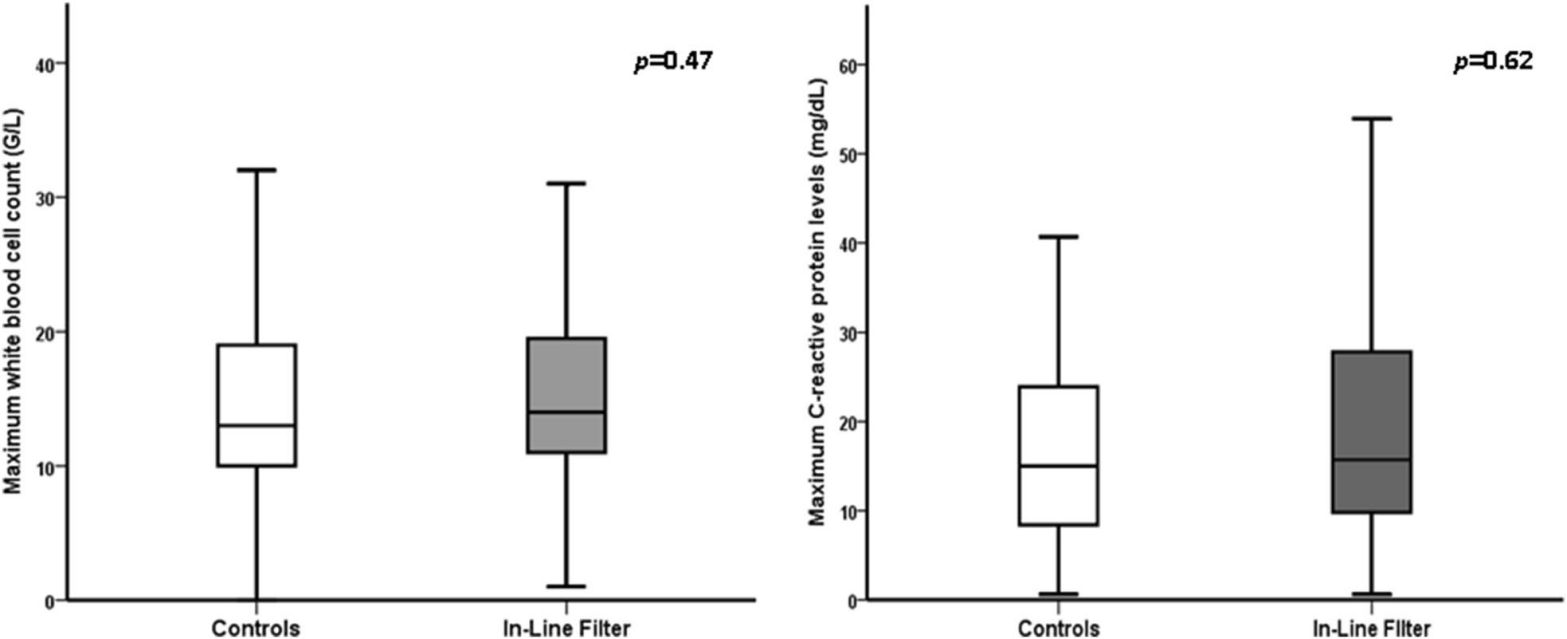
Differences in the maximum white blood cell count and C-reactive protein levels between study groups

Of 1716 patients screened for study eligibility, 504 patients were enrolled in the trial. No patient was lost to follow-up (Fig. 2). The study groups did not differ in demographic data, comorbidities, admission diagnoses or other clinical data (Table 1). There was no difference in the number of days in the ICU with SIRS between microfilter and control patients. Except for a higher incidence of SIRS in in-line microfilter patients, no difference between groups was observed in any secondary outcome parameter. The results did not change when only patients with an ICU length of stay of greater than 7 days were included in the analysis (Table 2). The maximum white blood cell count and maximum C-reactive protein serum concentrations did not differ between the groups (Fig. 3). The rate of adverse events was comparable between microfilter and control patients (Table 3). In two patients allocated to the in-line microfilter group, the study intervention was discontinued. In one patient, a leakage of propofol occurred from the in-line microfilter leading to inadequate sedation and patient-ventilator dyssynchrony. In the other patient, the three-way cock proximal of the in-line microfilter was missing and rescue fluids could only be administered at a too low velocity. The use of in-line microfilters in the study group resulted in additional median costs per patient of 54.7 (54.7–109.4) € and 25 544.9 € for the entire study group.

**Table 1:** Patient characteristics

	In-line filter group	Control group	*p* value
*n*	252	252	
Age (years)	66.5 (56–76)	68 (58–77)	0.33
Male gender (*n*/%)	151 (59.9)	145 (57.5)	0.65
BMI (kg/m^2^)	26 (23–30)	25 (23–29)	0.35
Comorbidities (*n*/%)			
Chronic arterial hypertension	101 (40.1)	96 (38.1)	0.71
Coronary artery disease	36 (14.3)	25 (9.9)	0.17
Congestive heart failure	31 (12.3)	26 (10.3)	0.57
COPD	50 (19.8)	46 (18.3)	0.73
Diabetes mellitus	32 (12.7)	35 (13.9)	0.79
Malignancy	58 (23.0)	53 (21.0)	0.67
Admission diagnoses (*n*/%)			
Abdominal surgery	72 (28.6)	62 (24.6)	0.36
Cardiovascular surgery	40 (15.9)	43 (17.1)	0.81
Trauma	26 (10.3)	25 (9.9)	1
Other surgery	52 (20.6)	48 (18.3)	0.74
Medical condition	62 (24.6)	76 (30.1)	0.19
Blood transfusion (*n*/%)	86 (34.1)	78 (31.0)	0.51
Vasopressor therapy (*n*/%)	163 (64.7)	158 (62.7)	0.71
Invasive mechanical ventilation (*n*/%)	123 (48.8)	114 (45.2)	0.47
Replacement therapy (*n*/%)	24 (9.5)	26 (10.3)	0.88
ECMO therapy (*n*/%)	5 (2.0)	5 (2.0)	1.00

**Table 2:** Primary and secondary outcome parameters

	In-line microfilter group	Control group	*p* value
All patients			
*n*	252	252	
Primary outcome parameter			
Days with SIRS (days)	2 (0.8–4.7)	1.8 (0.7–4.4)	0.62
Secondary outcome parameters			
Patients with SIRS (*n*/%)	251 (99.6)	244 (96.8)	0.04*
SIRS criteria per SIRS day (*n*)	2 (2–2)	2 (2–3)	0.77
New ALI/ARDS (*n*/%)	8 (3.2)	6 (2.4)	0.77
Duration of invasive mechanical ventilation (days)	0.5 (0.1–2)	0.8 (0.3–2.7)	0.14
Length of stay in the ICU (days)	2.3 (1–5.2)	2 (1–4.7)	0.53
ICU mortality (*n*/%)	30 (11.9)	28 (11.1)	0.89
Post hoc analysis: ICU stay > 7 days			
*n*	37	35	
Days with SIRS (days)	8 (6–16)	9 (6–17)	0.76
Patients with SIRS (*n*/%)	37 (100)	35 (100)	1
SIRS criteria per SIRS day (*n*)	2 (2–3)	2 (2–3)	0.89
New ALI/ARDS (*n*/%)	7 (18.9)	4 (11.4)	0.52
Duration of invasive mechanical ventilation (days)	4 (1–10)	5 (3–9)	0.89
Length of stay in the ICU (days)	10 (8–17)	12 (9–19)	0.64
ICU mortality (*n*/%)	9 (24.3)	5 (14.3)	0.38

SIRS, systemic inflammatory response syndrome; ALI/ARDS, acute lung injury/acute respiratory distress syndrome; ICU, intensive care unit

* Significant difference between groups

**Table 3:** Adverse events

	In-line filter group	Control group	*p* value
*n*	252	252	
New candida bloodstream infection (*n*/%)	0 (0)	2 (0.8)	0.5
New central-line-associated bloodstream infection (*n*/%)	1 (0.4)	1 (0.4)	1
New thromboembolic event (*n*/%)	1 (0.4)	3 (1.2)	0.62
Patients with hyperglycemia (*n*/%)	109 (43.3)	121 (48)	0.33
Insulin therapy (*n*/%)	91 (36.1)	104 (41.3)	0.27
Cumulative insulin dose (IU)	58 (18–136)	53 (22–207)	0.95
Patients with hypoglycemia (*n*/%)	27 (10.7)	19 (7.5)	0.28

Hyperglycemia defined as serum glucose > 180 mg/dL at one or more measurements; hypoglycemia defined as serum glucose < 75 mg/dL at one or more measurements

## Discussion

In this randomized, controlled open-label trial, we could not confirm our hypothesis that in-line microfilters reduce the number of ICU days with SIRS in the adult critically ill patients. In addition, no differences in other markers of immune activation, the rate of acute lung injury or the acute respiratory distress syndrome, duration of mechanical ventilation, ICU length of stay, and ICU mortality were observed. The results did not change when including only patients with an ICU length of stay of greater than 7 days. Adverse events occurred at a similar rate in patients with and without in-line microfilter use. The use of in-line microfilters was associated with additional costs.

Similar to the study proving the beneficial effects of in-line microfilters in critically ill children [13], we used the incidence, duration and extent of SIRS as the main indicator of systemic immune activation and primary study endpoint in this study. Since three of four SIRS criteria were documented electronically at 1-min intervals, almost all study patients fulfilled the non-time-related SIRS criteria at least once during their ICU stay. Therefore, the very high rate of SIRS in this patient population is likely to have over-estimated the true incidence of SIRS due to systemic inflammation as some increases in heart and respiratory rate may have simply reflected physical activity or resulted from nursing care. As the serum concentrations of the C-reactive protein and the white blood cell count are typically not influenced by the above-mentioned factors, it seems prudent to use these parameters as a more reliable marker of immune activity than SIRS in this closely monitored patient population. Both the C-reactive protein and white blood cell count reflect systemic inflammation in the critically ill [19, 20].

Although the efficacy of in-line microfilters to prevent particles from entering the bloodstream was not tested in our study, several previous reports confirmed this ability of the microfilters used in our study [13–15]. Interestingly, despite the fact that one can assume that infusion of microparticles was reduced in the intervention group, no difference in clinical or laboratory signs of immune activation could be detected between patients with and without in-line microfilter care. Consequently, outcome parameters associated with systemic inflammation, such as acute lung injury, duration of mechanical ventilation, length of ICU stay and ICU mortality [21], were comparable between the study groups. As the number of ICU days with SIRS observed in this study population matched well with what the power analysis had been based on, it is unlikely that our trial was underpowered to detect a significant difference between groups in this study population. Our data suggest that filtration of particles from injections and infusions by in-line microfilters does not modulate the systemic immune response in the adult critically ill patients and cannot prevent acute lung injury or reduce the duration of mechanical ventilation. It can be debated what role particles played that were introduced into the bloodstream by rescue fluids administered proximal to the in-line microfilters.

Our results are in contrast to the observations made with the use of in-line microfilters in critically ill children, in whom beneficial effects on hematologic, renal and respiratory function were reported [14, 16]. Using a comparable study design, a randomized, controlled open-label trial including 807 pediatric ICU patients revealed that the use of in-line microfilters decreased the incidence of SIRS, the length of ICU stay, and the duration of mechanical ventilation [13]. Similar to our study, the same type of in-line microfilters, a standardized infusion regimen and identical standards of filter care were applied. These similarities in the design and intervention suggest that differences between ours and the pediatric studies could result from a different inflammatory response of critically ill children to particle infusion compared to adults. On the other hand, one needs to consider the possibility that the positive findings of the pediatric trial may have reflected a type I error given its single-center and unblinded design. Interestingly, a recent Cochrane meta-analysis on the use of intravenous in-line filters in neonates included four trials with a total of neonates. No difference in overall mortality, proven and suspect septicemia or other secondary outcomes such as local phlebitis, thrombosis, necrotizing enterocolitis, duration of cannula patency, length of hospital stay, number of catheters inserted and financial costs was detected [22]. Unlike the pediatric studies, we did not evaluate specific extra-pulmonary organ functions such as renal or hematologic function. Yet, it is unlikely that we have missed a clinically relevant difference in these organ functions as this would have probably affected the length of ICU stay or other outcome parameters [23].

The rate of adverse events was a secondary endpoint of this trial. Due to their physical ability, in-line microfilters may theoretically bind drugs such as heparins, insulin, and others. As no difference in the rate of new thromboembolic complications, glycemic derangements, and cumulative insulin requirements was observed between groups, it can be assumed that drug binding by in-line microfilters occurs, if at all, at a clinically irrelevant extent. On the other hand, our study failed to show any beneficial effects which could result from the use of in-line microfilters as for example filtering of bacteria and yeasts. Accordingly, the rate of central-line-associated bloodstream infections and new candidemia was comparable between the study groups.

Certain limitations need to be considered when interpreting the results of this study. We applied a non-blinded design as we did not use sham filters. Even though voluntary contamination of injections or infusions with particles is unlikely to have occurred, we cannot exclude that a risk of bias has been introduced by choosing an unblinded design [24]. Another limitation is that we selected 504 out of 1716 patients screened. An expected ICU stay <24 h and no central venous catheter in place were the most common reasons for exclusion. Therefore, the results of our study cannot be extrapolated to patients with an ICU stay <24 h. Finally, this trial was conducted in a single center and included a mixed critically ill adult patient population. We do not know whether different results would have been observed had the trial been performed in several centers or had only selected patient populations been included [25].

## Conclusions

In conclusion, the use of in-line microfilters failed to modulate systemic inflammation and clinical outcome parameters in adult critically ill patients.

## Abbreviations

ICU: intensive care unit
SIRS: systemic inflammatory response syndrome
